# Flow Cytometric Immunophenotyping: Minimal Differences in Fresh and Cryopreserved Peripheral Blood Mononuclear Cells versus Whole Blood

**DOI:** 10.3390/biomedicines12102319

**Published:** 2024-10-11

**Authors:** Andrea Tompa, Junko Johansson, Ulrika Islander, Maria Faresjö

**Affiliations:** 1 Department of Clinical Diagnostics, School of Health and Welfare, Jönköping University, SE-551 11 Jönköping, Sweden; andrea.tompa@ju.se; 2 Division of Systems and Synthetic Biology, Department of Life Sciences, Chalmers University of Technology, SE-412 96 Gothenburg, Sweden; junko@chalmers.se (J.J.); islander@chalmers.se (U.I.); 3 Department of Rheumatology and Inflammation Research, Institute of Medicine, Sahlgrenska Academy, University of Gothenburg, SE-405 30 Gothenburg, Sweden

**Keywords:** cytometry, peripheral blood mononuclear cells, cryopreservation, whole blood, immunophenotyping

## Abstract

**Background/Objectives**: Flow cytometry is a convenient tool in immunophenotyping for monitoring the status of immunological conditions and diseases. The aim of this study was to investigate the effect of isolation and cryopreservation by flow cytometric analysis on subpopulations of CD4^+^ T helper (Th), T regulatory (Treg), CD8^+^ T cytotoxic (Tc), CD56^+^ NK, CD19^+^ B and monocytes. Freshly isolated and cryopreserved peripheral blood mononuclear cells (PBMCs) were compared to fresh whole blood. **Methods**: Peripheral blood was collected from healthy donors and prepared for flow cytometric analysis using the same panels of antibodies throughout the study. **Results**: Comparisons between fresh (F)- and cryopreserved (C)-PBMCs showed no major differences in percentages of CD4^+^, Th1, Th2 and CD4^+^CD25^+^CD127^low^ Treg cells. No differences in percentage of CD8^+^ or subpopulations of naive/stem, central or effector memory cells were observed between F- and C-PBMCs. The percentage of CD56^+^ NK cells, CD19^+^ B cells or classical and nonclassical monocytes did not differ between F-and C-PBMCs either. On the contrary, whole blood had lower percentages of Th and NK cells but higher percentages of Th1, Th17, Th1Th17, Tregs, Tc and B cells compared to C-PBMCs, while it had a higher proportion of Tc compared to F-PBMCs. **Conclusions**: Flow cytometric immunophenotyping minimally differs between freshly isolated and cryopreserved PBMCs. This implies the possibility of cryostorage of cohorts for later analysis. Importantly, care must be taken when comparing results from whole blood with isolated and cryopreserved PBMCs. Collectively, these results can contribute to the standardization of flow cytometric protocols in both clinical and research settings.

## 1. Introduction

Flow cytometry is a versatile technology widely used in both clinical practice and in various research fields. Immunophenotyping of blood with flow cytometry, wherein the different immune cell populations are characterized, is used clinically to diagnose and monitor the status of many immunological conditions and diseases, such as inherited immunodeficiencies and hematological cancers [1]. The research applications are numerous and include everything from schizophrenia to nanomaterials [2,3].

Due to constraints and limitations in sampling and availability, blood collected for research purposes is, by necessity, often not gathered at a single time point but rather during a period of time. Samples can be analyzed at each separate time point, either with intact peripheral whole blood or freshly isolated peripheral blood mononuclear cells (PBMCs), or with PBMCs cryopreserved in a biobank for simultaneous analysis of multiple samples at a later time point. Batch analysis facilitated by the usage of cryopreserved PBMCs negates the introduction of variability caused by analyses performed on different days, with different instrumental settings or by different operators. Cryopreservation also opens up the possibility for multiple centers and studies to use the same sample set, thus optimizing the use of limited resources.

However, cryopreservation also has several disadvantages. It necessitates multiple technical steps that might affect the cells of interest and can introduce several artifacts into the subsequent analysis. Isolation from whole blood, freezing and thawing have been shown to affect immune cells by altering, e.g., the fraction of different subpopulations and the expression of various markers [4,5,6,7,8,9,10,11,12]. By instead utilizing fresh whole blood for flow cytometry analysis, the effects of isolation and storage of the cells are removed. It is also much more time efficient to use whole blood, since the sample preparation is not as extensive. Although, if the immune cell population of interest is rare and present at low numbers in the blood, it might be beneficial to perform an isolation step to separate PBMCs from other blood components.

Our group has previously investigated the effects of isolation and cryopreservation/storage on immune cells by flow cytometric analysis of peripheral blood. These studies have shown that, while freshly isolated PBMCs and cryopreserved PBMCs did not differ in the fraction of CD19^+^ B cells and CD16^+^CD56^+^ NK cells when analyzed by flow cytometry, whole blood had a higher proportion of B cells and lower proportion of NK cells than both PBMC sample groups. Furthermore, while the percentage of total CD3^+^ T cells was the same regardless of whether or not the samples had been isolated and cryopreserved, whole blood showed an alternate pattern in the fraction of CD4^+^ T helper cells and CD8^+^ T cytotoxic cells compared to isolated PBMCs. It was also shown that freshly isolated PBMCs and cryopreserved PBMCs had very similar distributions of T cell subpopulations, such as CD4^+^CD25^+^CD127^−^ cells with a T regulatory (Treg) phenotype [13,14].

The first aim of this study was to investigate the effect of isolation and cryopreservation on different subsets of PBMCs, with a focus on T cells, by flow cytometric analysis. The secondary aim was to compare the proportion of isolated subsets of PBMCs, either fresh or cryopreserved, with fresh whole blood. A wide range of subpopulations of CD4^+^ T helper (Th) cells were analyzed, including cells that phenotypically can be classified as Th1 cells (CD4^+^CD45RO^+^CCR4^−^CCR6^−^CXCR3^+^), Th2 (CD4^+^CD45RO^+^CCR4^+^CCR6^−^CXCR3^−^), Th17 (CD4^+^CD45RO^+^CCR4^+^CCR6^+^CXCR3^−^), Th1Th17 (CD4^+^CD45RO^+^CCR4^−^CCR6^+^CXCR3^+^) [15,16,17] and T regulatory (Treg) (CD4^+^CD25^+^CD127^low^ or CD4^+^CD25^+^CD127^low^FoxP3^+^) cells [18]. The CD8^+^ T cytotoxic (Tc) cells were divided into naive/stem cell memory cells (CD8^+^CD45RA^+^CCR7^+^), central memory (CD8^+^CD45RA^−^CCR7^+^), effector memory (CD8^+^CD45RA^−^CCR7^−^) and CD45RA^+^ effector memory cells (TEMRA, CD8^+^CD45RA^+^CCR7^−^) [19,20]. While only the main B cell (CD3^−^CD19^+^) population was analyzed, NK cells (CD3^−^CD56^+^) were divided into two subpopulations for further investigation of the more cytotoxic CD56^dim^CD16^+^ cells and the cytokine-producing CD56^bright^CD16^−^ cells [21,22]. Finally, the analysis was performed on classical (CD14^+^CD16^−^), intermediate (CD14^+^CD16^+^) and nonclassical (CD14^−^CD16^+^) monocytes [23]. The samples were all analyzed with the same panels of antibodies against numerous lymphocyte and monocyte subpopulations.

The outcome of this study can potentially contribute to the standardization of flow cytometric protocols in both clinical practice and in various research fields.

## 2. Materials and Methods

### 2.1. Study Population

To evaluate the effect of isolation and cryopreservation on immune cells and their subpopulations, flow cytometric phenotyping was performed on whole blood samples, freshly isolated PBMCs and cryopreserved PBMCs, thawed one week after cryopreservation.

Blood samples were collected by venipuncture from a total of 10 blood donors (5 male, 5 females, 18–65 years of age) who donate blood regularly at the Department of Transfusion Medicine, Region Jönköping County, Jönköping, Sweden.

### 2.2. Sample Collection and Isolation of PBMCs

From each donor, totally ca 35 mL of venous blood were collected in one BD Vacutainer K_2_-EDTA tube (BD Biosciences, San Jose, CA, USA) and four BD Vacutainer (BD Biosciences) Cell Preparation Tubes (CPTs) supplemented with sodium-heparin. The blood samples collected in CPTs were used for the isolation of PBMC, as previously described [14]. In accordance with the manufacturer’s instructions, the blood samples were processed within 2 h after sampling. Briefly, well-mixed blood samples in CPTs were centrifuged for 20 min at 1500× *g* at room temperature. After centrifugation, the collected cell suspension was washed twice in phosphate-buffered saline (PBS), pH 7.4 (Gibco, Darmstadt, Germany), supplemented with 2% fetal calf serum (FCS, Gibco). The cells were finally resuspended in 1 mL cell medium (RPMI-1640 medium (Sigma-Aldrich AB, Stockholm, Sweden)) supplemented with 2% FCS.

### 2.3. Cryopreservation and Thawing of PBMCs

For cryopreservation, 4 °C freezing media (consisting of 10% dimethyl sulfoxide (Sigma-Aldrich AB), 40% FCS and 50% RPMI-1640) was added to cell suspensions dropwise under continuous mixing. One milliliter aliquots, i.e., 5 × 10^6^ PBMCs/mL in cryotubes (Nalgene^®^, VWR International, Bristol, UK), were placed into a freezing container ‘MrFrosty’ (Nalgene^®^) and kept overnight at −80 °C before being transferred to −150 °C for storage.

Cryopreserved PBMCs were thawed in a 37 °C water bath by gentle continuous mixing. The cell suspension was resuspended in cell wash medium (PBS with 2% FCS) and washed twice and finally resuspended in 1 mL RPMI 1640 medium with 2% FCS.

After thawing, all PBMC samples were allowed to rest at room temperature for 1 h before staining with fluorochrome-conjugated monoclonal antibodies in advance of the flow cytometric analysis.

### 2.4. Flow Cytometry

#### 2.4.1. Staining of Immune Cells and Data Collection

Monoclonal antibodies purchased from BD Biosciences were used for the determination of different cell population frequencies in whole blood, fresh and cryopreserved PBMCs. The combination of antibodies used for the staining of surface markers in all samples are presented in Table 1.

The staining and analysis of whole blood were performed within 4 h of sample collection; therefore, viability of the immune cells in whole blood was not measured. The cell viability of the PBMC samples was determined with a TC20 automated cell counter (Bio-Rad Laboratories, Hercules, CA, USA) via trypan blue exclusion. The mean cell viability in fresh PBMC samples was ≥95% and ≥90% in cryopreserved samples.

The immune cells in whole blood and PBMCs were stained with fluorochrome-conjugated monoclonal antibodies. Then, 50 μL BD Horizon™ Brilliant Stain buffer (BD Biosciences) and 100 μL whole blood or PBMC samples (approximately 2.5 × 10^5^ immune cells) were added to each staining tube. For staining of the extracellular markers, titrated amounts of fluorochrome-conjugated monoclonal antibodies were added to the whole blood and PBMC samples, respectively, and incubated for 30 min at room temperature, protected from light. After incubation, the erythrocytes in the whole blood samples were lysed by adding 2 mL FACS™ Lysing Solution (BD Biosciences), followed by another incubation for 15 min. Thereafter, the whole blood samples were washed with PBS and resuspended in 500 μL of PBS. The PBMC samples were washed directly after incubation with PBS and resuspended in 500 μL of PBS.

After completed extracellular marker staining, the cells in all sample types were permeabilized for staining of the intracellular markers. Fixation and permeabilization of the cells were performed with a BD Pharmingen™ Transcription Factor Buffer Set (BD Biosciences), in accordance with the manufacturer’s instructions. Following the extracellular staining, one milliliter of freshly prepared Fix/Perm buffer (working solution) was added to each tube, vortexed and incubated at 2–8 °C for 30–40 min, protected from light. Following fixation and permeabilization, one milliliter of Perm/Wash buffer (working solution) was added to the cells. After centrifugation (6 min at 350× *g* at 2–8 °C), the supernatant was aspirated. Thereafter, the cells were washed by adding 2 mL of Perm/Wash buffer (working solution) and centrifuged for 6 min at 350× *g* at 2–8 °C. Finally, the cells were resuspended in 200 µL of Perm/Wash buffer (working solution). Fluorochrome-conjugated monoclonal antibodies specific for intracellular proteins (FoxP3 and perforin) were added to each tube, vortexed and incubated at 2–8 °C for 30–40 min, protected from light. Post-incubation, the cells were briefly vortexed and washed with 2 mL of Perm/Wash buffer, followed by centrifugation. Finally, the cells were resuspended in 400 µL of flow cytometry stain buffer.

Acquisition of data on the flow cytometer was performed within 2 h after staining. All flow cytometric data were acquired on a BD FACSCanto II Flow cytometer (BD Biosciences) with software BD FACSDiva™ 8.0. The acquisition gates were restricted to lymphocyte gates based on morphological characteristics, and a minimum of 100,000 lymphocytes were acquired and analyzed.

#### 2.4.2. Gating Strategy and Analysis of Data

The data analyses were performed with Kaluza Analysis 2.2 software (Beckman Coulter, Indianapolis, IN, USA) by one person to avoid inter-assay variation. Lymphocytes and monocytes were identified based on their size in forward scatter (FSC) and morphological characteristics on side scatter (SSC). Doublet cells and debris were excluded from the analysis using FSC height and area.

#### 2.4.3. Quality Control

Sample acquisition was performed in a total of nine experimental runs performed during a time period of 15 consecutive days. A biological control was used in every flow cytometric run to check the experimental stability. The biological control consisted of PBMCs isolated from whole blood that had been frozen in multiple aliquots. The controls were stained with antibodies for T, Th, Tc, B and NK cells. Evaluation of the results from the biological controls shows stability and reproducibility across all flow cytometric runs (Supplementary Figure S1A). The estimated inter-assay coefficients of variation (CV) for the biological controls are below the acceptable limits (<10%) (Supplementary Figure S1B) [24].

The instrument’s performance was checked daily using the set-up and tracking application BD FACS 7-Color Set-up Beads (BD Biosciences) and BD CST (BD Biosciences). The same lots of monoclonal antibodies were used throughout the study. Compensation beads (BD Biosciences) were used to optimize fluorescence compensation settings for multicolor flow cytometric analysis. Fluorescence minus one (FMO) control (samples that contain all the antibodies in a panel, minus one of them, while the others remain constant) were used to ensure proper gate settings [25]. All antibodies were titrated to achieve the highest optical signal for the positive population and the lowest signal for the negative population, representing the optimal signal-to-noise ratio (Table 1) [25].

### 2.5. Statistical Analysis

Statistical analyses were performed in GraphPad Prism 10 (GraphPad Software, Boston, MA, USA). All data for comparisons of median values between groups were analyzed with non-parametric, paired Friedman tests, followed by Dunn’s multiple comparisons tests. The resulting *p*-values are automatically corrected for multiple testing through multiplication with the number of groups in each analysis [26]. The adjusted *p*-values are subsequently compared to the α-threshold of 0.05 in order to determine statistical significance. Correlation coefficients were calculated with non-parametric Spearman correlation.

### 2.6. Ethics

The blood donors were informed orally and invited to voluntary participation in the study. Blood samples were collected and directly unidentified at the same time as blood was drawn for blood banking. Ethical approval was not applied, since the collected samples, as well as the results, cannot be derived back to individuals, which is in concordance with the Swedish statute book (2003:460;3).

## 3. Results

### 3.1. CD4^+^ T Helper Cells and T Regulatory Cells

**Figure 1:**
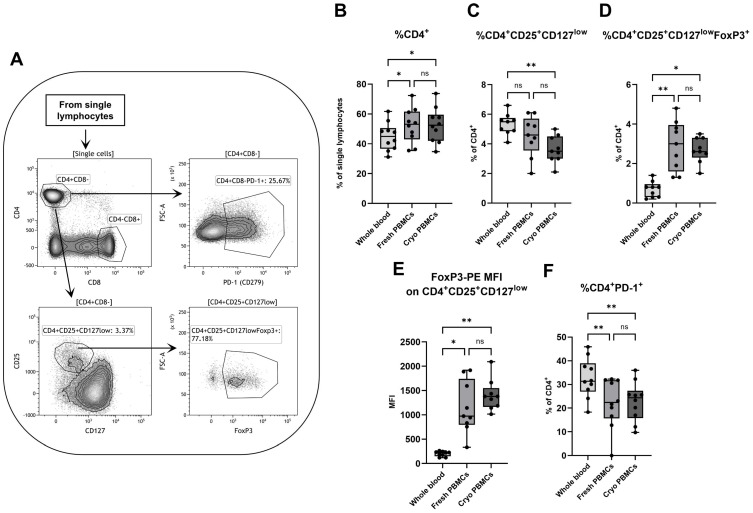
Flow cytometry analysis of CD4^+^ T helper cells, with (**A**) a representative gating strategy for a PBMC sample. The percentages of (**B**) CD4^+^ T cells among single lymphocytes were analyzed by flow cytometry, as well as the proportions of (**C**) CD25^+^CD127^low^, (**D**) CD25^+^CD127^low^FoxP3^+^ and (**F**) PD-1^+^ cells among all CD4^+^ T cells. (**E**) The FoxP3 MFI for the CD25^+^CD127^low^ cells was also measured. PBMCs = peripheral blood mononuclear cells. Cryo = cryopreserved. MFI = median fluorescence intensity. ns=non-significant. Paired non-parametric Friedman test, followed by Dunn’s multiple comparisons test; n = 10 for (**B**,**F**) and n = 9 for (**C**–**E**). * indicates *p* < 0.05 and ** *p* < 0.01 (adjusted *p*-values).

To investigate the effect of isolation and cryopreservation on CD4^+^ T helper cells, a gating strategy was utilized wherein CD4^+^ T helper cells were separated from CD8^+^ T cytotoxic cells and further analyzed for the presence of other markers in order to identify various subpopulations (Figure 1A). While there was no difference in percentage of CD4^+^ T helper cells among all lymphocytes between fresh and cryopreserved PBMCs, whole blood contained less CD4^+^ T helper cells compared to both freshly isolated and cryopreserved PBMC samples (Figure 1B). This is reflected in the correlation coefficients, where the percentage of CD4^+^ T helper cells are high for each possible combination between the three groups but are the lowest for comparisons between whole blood and both PBMC sample groups (Supplementary Figure S2A).

Tregs were characterized in two ways; through the simultaneous expression of CD25 with CD127 (CD4^+^CD25^+^CD127^low^) or by the latter with the added expression of intracellular FoxP3 (CD4^+^CD25^+^CD127^low^FoxP3^+^). When defined by only CD25 and CD127, whole blood contained a higher percentage of Tregs (CD25^+^CD127^low^) compared to cryopreserved PBMCs (Figure 1C). This was not the case when the intracellular expression of FoxP3 was added, since the proportion of these Tregs (CD25^+^CD127^low^FoxP3^+^) was lower in whole blood compared to both freshly handled and cryopreserved PBMCs (Figure 1D). Interestingly, the median fluorescence intensity (MFI) of FoxP3 in whole blood was lower for both the CD4^+^CD25^+^CD127^low^ population (Figure 1E) and the main CD4^+^ T helper and CD8^+^ T cytotoxic cell populations (Supplementary Figure S3A,B).

Fresh and cryopreserved PBMCs did not differ in the percentage of CD4^+^PD-1^+^ T cells, but both groups had lower percentages of CD4^+^PD-1^+^ cells compared to whole blood (Figure 1F). This was also reflected by higher PD-1 MFI expression on CD4^+^ T helper cells in whole blood compared to cryopreserved PBMCs. There was no discernible difference in PD-1 MFI expression for the CD25^+^CD127^low^ T cells and the CD8^+^ T cells (Supplementary Figure S3C–E).

The MFI values for CD26, CD122 and CD127 were also analyzed. The expression differs in different groups, depending on the marker and analyzed population; see Supplementary Table S1 for detailed information.

### 3.2. CD4^+^ T Helper Cell Subpopulations

**Figure 2:**
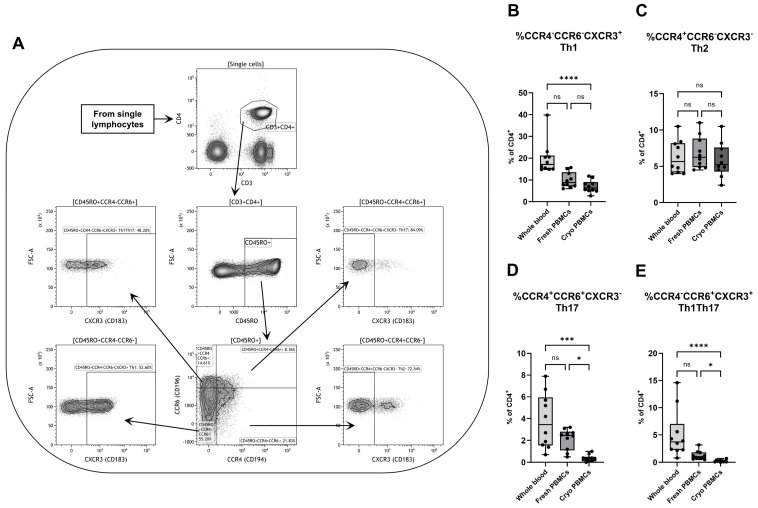
Flow cytometry analysis of different CD4^+^ T helper subpopulations with (**A**) a representative gating strategy for a PBMC sample. The percentages of CD4^+^ T cells with (**B**) Th1 (CCR4^−^CCR6^−^CXCR3^+^), (**C**) Th2 (CCR4^+^CCR6^−^CXCR3^−^), (**D**) Th17 (CCR4^+^CCR6^+^CXCR3^−^) and (**E**) Th1Th17 (CCR4^−^CCR6^+^CXCR3^+^) phenotypes were analyzed by flow cytometry. PBMCs = peripheral blood mononuclear cells. Cryo = cryopreserved. ns = non-significant. Paired non-parametric Friedman test, followed by Dunn’s multiple comparisons test, n = 10. * Indicates *p* < 0.05, *** *p* < 0.001 and **** *p* < 0.0001 (adjusted *p*-values).

To further investigate the effect on various CD4^+^ subpopulations, cells were gated on their expression of the chemokine receptors CCR4 (CD194), CCR6 (CD196) and CXCR3 (CD183). After a first gating on CD45RO, CD4^+^ T cells were defined according to their simultaneous expression of CCR4 and CCR6 to subsequently be gated for the expression of CXCR3 (Figure 2A). Due to the continuous expression of CCR4 and CCR6, the gates for these receptors were based on the FMO control and were therefore the same for all samples in all three groups. CXCR3 had a clear distinction between cells with positive and negative expressions, and the gating was manually defined and slightly shifted between different samples when needed.

While there were no differences between fresh and cryopreserved PBMCs in percentages of cells with a Th1-like phenotype, here defined as CD4^+^CD45RO^+^CCR4^−^CCR6^−^CXCR3^+^, the percentage in whole blood was higher than in cryopreserved PBMCs (Figure 2B). Both PBMC groups and whole blood had similar proportions of Th2-like cells (CD4^+^CD45RO^+^CCR4^+^CCR6^−^CXCR3^−^) (Figure 2C). Cryopreserved PBMCs had a lower percentage of cells with a Th17 phenotype (CD4^+^CD45RO^+^CCR4^+^CCR6^+^CXCR3^−^) compared to both fresh PBMCs and whole blood, with the latter containing the highest proportion (Figure 2D). Corresponding to the results for Th1 and Th17, whole blood had the highest fraction of cells with a phenotype similar to Th1Th17 cells (CD4^+^CD45RO^+^CCR4^−^CCR6^+^CXCR3^+^) (Figure 2E). To solidify the findings, a correlation matrix was created, with the fraction of Th1-like cells as an example population. Fresh and cryopreserved PBMCs have low correlation coefficients in comparison to whole blood but much higher coefficients in comparison to each other (Supplementary Figure S2B).

The MFI values for the chemokine receptors were also analyzed for both the main CD4^+^ population and the CD4^+^CD25^+^CD127^low^ cells. These values, as well as the expression of CCR5 and CCR10 on the main CD4^+^ and CD8^+^ T cell populations, can be found in Supplementary Table S1.

### 3.3. CD8^+^ T Cytotoxic Cells

**Figure 3:**
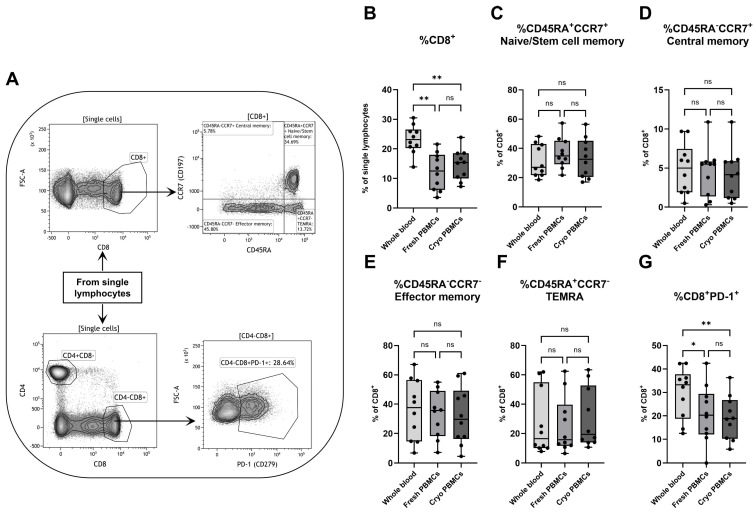
Flow cytometry analysis of CD8^+^ T cytotoxic cells with (**A**) a representative gating strategy for a PBMC sample. The percentages of (**B**) CD8^+^ T cells among single lymphocytes were analyzed by flow cytometry, as well as the proportions of (**C**) naive/stem cell memory cells (CD45RA^+^CCR7^+^), (**D**) central memory (CD45RA^−^CCR7^+^), (**E**) effector memory (CD45RA^−^CCR7^−^), (**F**) TEMRA (CD45RA^+^CCR7^−^) and (**G**) PD-1^+^ cells among all CD8^+^ T cells. PBMCs = peripheral blood mononuclear cells. Cryo = cryopreserved. ns=non-significant. Paired non-parametric Friedman test, followed by Dunn’s multiple comparisons test, n = 10. * Indicates *p* < 0.05 and ** *p* < 0.01 (adjusted *p*-values).

Different memory subpopulations of CD8^+^ T cytotoxic cells were analyzed through the simultaneous expression of CD45RA and CCR7 (Figure 3A). While whole blood showed a higher percentage of total CD8^+^ T cells among all lymphocytes compared to both fresh and cryopreserved PBMCs (Figure 3B), no differences could be found for the different subpopulations of CD8^+^ T cells. Fresh and cryopreserved PBMCs had similar proportions as whole blood of naive/stem cell memory cells (CD8^+^CD45RA^+^CCR7^+^) (Figure 3C), central memory cells (CD8^+^CD45RA^−^CCR7^+^) (Figure 3D), effector memory cells (CD8^+^CD45RA^−^CCR7^−^) (Figure 3E) and of CD45RA^+^ effector memory cells (CD8^+^TEMRA, CD45RA^+^CCR7^−^) (Figure 3F).

The expression of PD-1 was analyzed as well, with whole blood containing a higher fraction of CD8^+^PD-1^+^ cells compared to both groups of PBMCs (Figure 3G). Additionally, the MFI values for CD39 and intracellular perforin staining were also analyzed and showed that, while all CD8^+^ memory subpopulations had similar distributions of CD39 for all three groups, the expression of perforin somewhat differed between different cell types and groups (Supplementary Table S1). In general, the intracellular expression of perforin was lower in whole blood compared to both groups of PBMCs, including all four memory subpopulations.

### 3.4. B Cells, NK Cells and Monocytes

**Figure 4:**
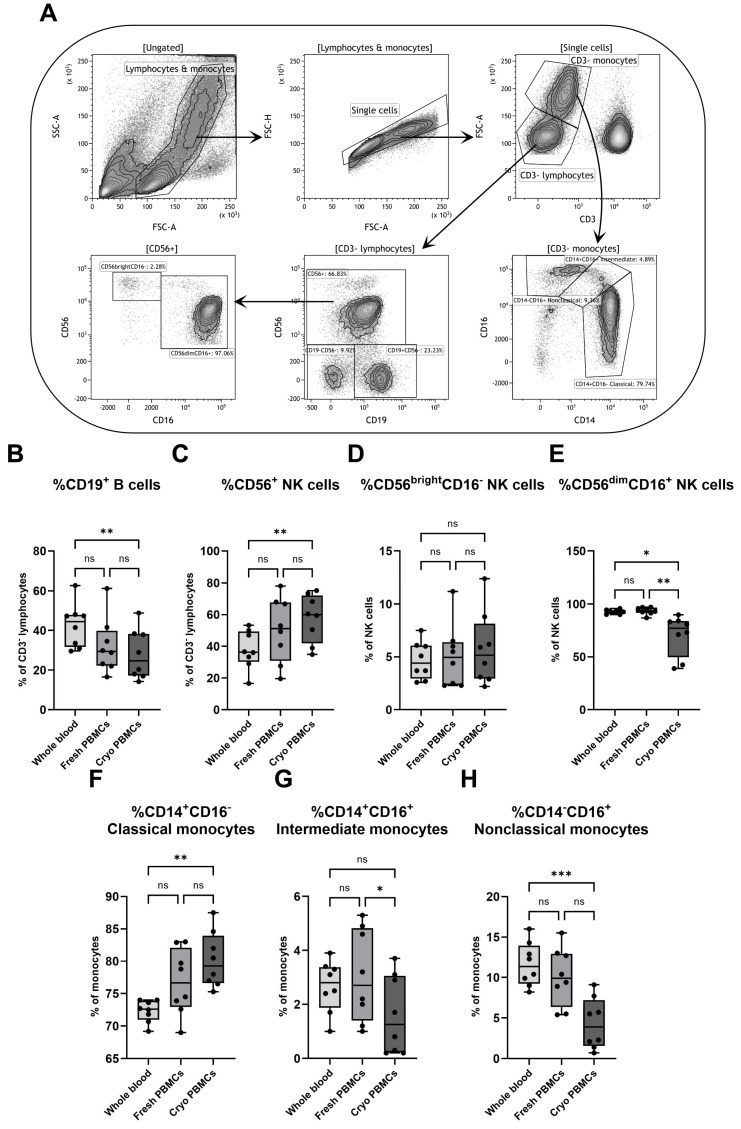
Flow cytometry analysis of various lymphocyte and monocyte populations with (**A**) a representative gating strategy for a PBMC sample. Flow cytometry was used to analyze the percentages of (**B**) CD19^+^ B cells and (**C**) CD56^+^ NK cells among all CD3^−^ lymphocytes, as well as the proportions of (**D**) CD56^bright^CD16^−^ and (**E**) CD56^dim^CD16^+^ NK cells and the percentages of (**F**) classical (CD14^+^CD16^−^), (**G**) intermediate (CD14^+^CD16^+^) and (**H**) nonclassical (CD14^−^CD16^+^) monocytes. PBMCs = peripheral blood mononuclear cells. Cryo = cryopreserved. ns = non-significant. Paired non-parametric Friedman test, followed by Dunn’s multiple comparisons test, n = 8. * Indicates *p* < 0.05, ** *p* < 0.01 and *** *p* < 0.001 (adjusted *p*-values).

The effect of isolation and cryopreservation was also investigated on other lymphocytes and on myeloid cells. After an initial gating on the negative expression of CD3, the cells were divided into two groups based on size, where the smaller population was denoted CD3^−^ lymphocytes and the larger population was characterized as monocytes. The lymphocytes were thereafter separated into B cells and NK cells based on the expression of CD19 and CD56, respectively, with the NK cells being further characterized with the addition of CD16 (Figure 4A).

While fresh and cryopreserved PBMCs did not differ in the percentage of B cells (CD3^−^CD19^+^), the fraction in whole blood was higher compared to cryopreserved PBMCs (Figure 4B). In contrast, cryopreserved PBMCs showed higher percentages of NK cells (CD3^−^CD56^+^) than whole blood (Figure 4C). For the two NK cell subpopulations, defined as CD56^bright^CD16^−^ and CD56^dim^CD16^+^ cells, respectively, no differences could be seen for the three groups in the CD56^bright^CD16^−^ subpopulation. However, cryopreserved PBMCs showed a lower fraction of CD56^dim^CD16^+^ cells compared to both fresh PBMCs and whole blood (Figure 4D,E).

Monocytes were further divided by the simultaneous expression of CD14 and CD16 into classical (CD14^+^CD16^−^), intermediate (CD14^+^CD16^+^) and nonclassical (CD14^−^CD16^+^) monocytes (Figure 4A). While whole blood contained a lower fraction of classical monocytes than cryopreserved PBMCs, it also had a higher percentage of nonclassical monocytes (Figure 4F,H). The two PBMC populations did not differ in percentage of classical and nonclassical monocytes, but cryopreserved PBMCs had a lower fraction of intermediate monocytes than fresh PBMCs. Interestingly, the fraction of intermediate monocytes did not differ between whole blood and the two PBMC groups (Figure 4G). The findings were solidified by correlation analyses, with the fraction of classical monocyte as a representative population. The correlation coefficients between whole blood and both PBMC sample groups were rather low but with higher values for comparisons between the two PBMC groups (Supplementary Figure S2C).

The MFI values of CD1a, CD11c and CD123 were also analyzed on both B cells and on the three monocyte populations and can be found in Supplementary Table S2.

## 4. Discussion

Our data show that the process of cryopreservation of isolated PBMCs does not seem to influence the major immune cell populations, as determined by flow cytometry. Comparisons between fresh and cryopreserved PBMCs show no major differences in the percentages of CD4^+^ T helper cells and CD8^+^ T cytotoxic cells, which is in accordance with previous studies [6,11,27], though there have also been reports of lower fractions of CD4^+^ T cells [12] and of both lower and higher proportions of CD8^+^ T cells [4,12,28,29] in cryopreserved PBMCs. Similar to other studies, we noticed no differences in the percentage of CD4^+^CD25^+^CD127^low^ Tregs between fresh and cryopreserved PBMCs [10,30]. No differences were seen for CD4^+^ T cells with Th1- (CCR4^−^CCR6^−^CXCR3^+^) and Th2 (CCR4^+^CCR6^−^CXCR3^−^)-like phenotypes, though cryopreserved PBMCs had lower proportions of both cells with Th17 (CCR4^+^CCR6^+^CXCR3^−^) and Th1Th17 (CCR4^−^CCR6^+^CXCR3^+^) phenotypes. Similar to our results, a study that utilized slightly different definitions of the Th subpopulations also showed no differences in fresh and cryopreserved PBMCs for Th1 and Th2 cells, defined as CCR4^−^CXCR3^+^CCR5^+^ and CCR5^−^CXCR3^−^CCR4^+^, but they also had no change in the proportion of Th17 cells when defined as CCR6^+^CD161^+^ [30].

In accordance with earlier reports, our study showed that there were no differences in the percentage of CD56^+^ NK cells [27,28] between fresh and cryopreserved PBMCs, though it was also shown that the proportion of CD56^+^ NK cells both increased and decreased after cryopreservation [12,29]. While we could not observe any influence on the percentage of CD19^+^ B cells, also observed from at least one other group [12], previous studies showed both higher and lower proportions of CD19^+^ B cells in cryopreserved PBMCs [4,28,29].

Our data also show that cryopreservation did not seem to affect the percentage of CD14^+^CD16^−^ classical monocytes, while it decreased the percentage of CD14^+^CD16^+^ intermediate monocytes with a trend towards a decrease in the fraction of CD14^−^CD16^+^ nonclassical monocytes. Interestingly, both intermediate and nonclassical monocytes express CD16, while classical monocytes lack this expression. This could also be observed for the NK cells where the CD16-negative CD56^bright^CD16^−^ NK cell subpopulation did not differ between the two PBMC groups, while the CD16-positive CD56^dim^CD16^+^ subpopulation did decrease after cryopreservation. A suggested reason for the lower proportions of these populations in cryopreserved PBMCs is thus either a loss of CD16 or issues with the CD16 antibody staining.

Contrary to the few differences observed between fresh and cryopreserved PBMCs, flow cytometric analyses of whole blood showed very different results compared to both groups of isolated PBMCs. Whole blood had lower percentages of CD4^+^ T cells and higher percentages of CD8^+^ T cells compared to both fresh and cryopreserved PBMCs, which has also been previously shown. It was also observed that whole blood had higher fractions of CD4^+^CD25^+^CD127^low^ Tregs compared to both groups of PBMCs, as already observed [10]. Depending on the phenotypic definition of Tregs, our results show that whole blood either has a higher (when defined as CD4^+^CD25^+^CD127^low^) or a lower (CD4^+^CD25^+^CD127^low^FoxP3^+^) fraction of Tregs. For unknown reasons, FoxP3 had a very low fluorescence intensity in whole blood, even though the same antibody lot was utilized for all tested groups throughout the whole study. This might be due to issues with the permeabilization or antibody staining, since whole blood has a very different matrix compared to isolated PBMCs, which might influence the intracellular staining procedure. The same volume of fixation/permeabilization solutions were used for all three sample groups, which might have been needed to be adjusted for whole blood for optimal effect. In a previous study by our group, where the same FoxP3 antibody (same clone and same fluorochrome) was used for the analysis of Tregs, a similar trend was observed with a very low percentage of CD4^+^CD25^+^CD127^−^FoxP3^+^ cells in whole blood [14]. However, in that study, fresh and cryopreserved PBMCs had similar fractions of CD4^+^CD25^+^CD127^−^FoxP3^+^ cells as whole blood, which is not the current case. One difference between the previous and the current study is the usage of fixation/permeabilization solutions from two different companies. It has been shown that the amount of detected FoxP3 is highly dependent on the choice of antibodies and fixation/permeabilization buffer [31], which might explain the discrepancy in our two studies. Though, this is just a theoretical speculation, and supplemental experiments are needed for confirmation.

We could also observe that, while the percentages of Th2 cells were similar for whole blood, fresh and cryopreserved PBMCs, the fraction of Th1, Th17 and Th1Th17 cells were higher for whole blood compared to both groups of PBMCs studied. Since the characterization of all four Th subpopulations was based on the expression of the same chemokine receptors (CCR4, CCR6 and CXCR3), and no change was observed for the Th2 cells, the decrease in expression of CD markers for the other three populations might not be due to methodological reasons, e.g., loss of markers during the preparation of PBMCs. Speculatively, this may rather be due to an actual loss of Th1, Th17 and Th1Th17 cells. Interestingly, Th1 and Th17 cells have a biological relation not shared with Th2 cells, wherein the plasticity of Th17 cells leads them towards Th1 polarization and the formation of Th1Th17 cells that produce both IFN-γ, a Th1 cytokine, and IL-17, the defining cytokine for Th17 cells [32,33].

While a previous study showed that the percentage of naive CD45RA^+^CCR7^+^ CD8^+^ T cells was decreased in whole blood compared to fresh PBMCs [7], in our results, the whole blood samples had a similar distribution of CD8^+^ CD45RA^+^CCR7^+^ naive/stem cell memory, CD45RA^−^CCR7^+^ central memory, CD45RA^−^CCR7^−^ effector memory and CD45RA^+^CCR7^−^ TEMRA cells as both fresh and cryopreserved PBMCs (with no difference found between the two PBMCs groups either). It should be noted that both naive and stem cell memory T cell populations express CD45RA and CCR7, and since we did not have a further discriminating marker in the flow cytometry panel (e.g., CD95 or CXCR3) [34], naive CD8^+^ T cells cannot be differentiated from CD8^+^ stem cell memory T cells and are therefore reported as naive/stem cell memory.

Whole blood had a lower percentage of CD56^+^ NK cells compared to cryopreserved PBMCs but a higher percentage of CD19^+^ B cells compared to the same group. Similar trends were shown towards fresh PBMCs, which was confirmed in a previous study where it was shown that the proportion of CD19^+^ B cells in whole blood was lower compared to both fresh and cryopreserved PBMCs [5]. Another difference between whole blood and the PBMC groups was that, while fresh and cryopreserved PBMCs differed in the percentage of intermediate monocytes, no difference could be found for that subpopulation for whole blood, but both classical and nonclassical monocytes differed between whole blood and cryopreserved PBMCs.

To summarize, our flow cytometric analyses show that, while there are only minor differences in immune cell populations and expression between isolated fresh PBMCs and isolated cryopreserved PBMCs, the expression of immune markers in fresh whole blood differs from both groups of studied PBMCs. This is not unexpected, since whole blood, in addition to immune cells, also has many other constituents, such as other cells, proteins and lipids, which can all affect the staining and analysis procedure. It is, for example, known that autofluorescence occurs in whole blood due to the presence of, e.g., porphyrin present in the hemoglobin of red blood cells [35]. Due to the differences in matrix compositions between whole blood and isolated PBMC solutions, the staining procedure, such as the concentration of antibodies, needs to be adjusted, depending on the sample material. In our study, the same antibody concentrations were used for all three sample groups, which, for whole blood with a higher probability of unspecific binding, might need to be separately optimized.

Our study focused on the short-term cryopreservation of PBMCs, where samples were frozen for one week. We did not focus on the effect of long-term storage of cryopreserved PBMCs. However, we have previously shown that cryopreservation for 6 months is, in general, feasible for a subsequent flow cytometric analysis. We previously showed that CD4^+^ cells were, in general, less affected by isolation and cryopreservation than subsets of CD8^+^ cells [14]. Furthermore, Tregs, defined as CD4^+^CD25^high^ expressing CD101 and CD129, CD4^+^CD25^high^CD127^−^ and CD4^+^CD25^high^CD127^−^FoxP3^+^, respectively, remained stable after isolation and cryopreservation [14]. In real-life applications, e.g., for biobanking purposes, samples may be frozen for months up to years, and though cell viability in general does not seem to be affected by cryopreservation, the immunophenotype may change over time [36]. Care should thus always be taken when comparing results between different studies, since the storage times may differ. In addition, other factors such as choice of anticoagulant and processing times might influence the outcome, underlining the importance of proper documentation. The relatively small sample size employed in our study should be noted. Larger cohorts create a more solid foundation for robust statistical analyses and subsequent generalizations, and small but important variations might be more noticeable in larger sample materials.

The strength of our study is that we simultaneously analyzed whole blood, fresh PBMCs and cryopreserved PBMCs from the same donors with multiple panels covering many different immune cell subpopulations, which are comparisons seldom reported. We analyzed a broad range of various immune cell populations, including lymphocytes such as subsets of CD4^+^ T helper cells, CD8^+^ T cytotoxic cells and NK cells, as well as monocyte subpopulations, which cover many clinically relevant immunophenotypes. This has enabled us to provide an extensive report with the conclusion that, while immunophenotyping by flow cytometry does not majorly differ between isolated fresh PBMCs and isolated cryopreserved PBMCs, a benefit for the purpose of biobanking, care must be taken when comparing results from whole blood with data from isolated PBMCs. Collectively, these results demonstrate that cryopreservation does not significantly alter the percentages of an extensive range of immune cells, and thus, this study can contribute to the standardization of flow cytometric protocols in both clinical and research settings.

## Figures and Tables

**Table 1:** Monoclonal antibodies used for the staining and flow cytometric analysis of different peripheral immune cells and their subsets.

Cell Populations	Antibody *	Clone	Titration	Fluorochrome
**CD4^+^ T helper and T regulatory cells**	CD4	SK3	1:2	PerCP-Cy5.5
	CD25	M-A251	1:2	PE-Cy7
	CD26	M-A261	1:2	BV510
	CD122	Mik-β3	1:4	APC
	CD127	HIL-7R-M21	1:2	BV421
	FoxP3	236A/E7	1:1	PE
	PD-1 (CD279)	EH12.1	1:1	BB515
**CD4^+^ T helper cell subpopulations**	CD3	UCHT1	1:2	FITC
	CD4	RPA-T4	1:2	APC-Cy7
	CD25	M-A251	1:2	PE
	CD45RO	UCHL1	1:2	PerCP-Cy5.5
	CD127	HIL-7R-M21	1:4	Alexa Fluor 647
	CCR4 (CD194)	1G1	1:2	BV510
	CCR5 (CD195)	2D7	1:4	APC
	CCR6 (CD196)	11A9	1:4	PE-Cy7
	CCR10	1B5	1:1	PE
	CXCR3 (CD183)	1C6	1:2	BV421
**CD8^+^ T cytotoxic cells**	CD8	SK1	1:2	APC-H7
	CD25	M-A251	1:2	PE-Cy7
	CD26	M-A261	1:2	BV510
	CD39	TU66	1:2	FITC
	CD45RA	HI100	1:4	PerCP-Cy5.5
	CD122	Mik-β3	1:2	APC
	CD127	HIL-7R-M21	1:2	BV510
	CCR7 (CD197)	150503	1:2	Alexa Fluor 647
	FoxP3	236A/E7	1:1	PE
	PD-1 (CD279)	EH12.1	1:1	BB515
	Perforin	δG9	1:1	BV421
**B cells, NK cells and monocytes**	CD1a	HI149	1:2	APC
	CD3	SK7	1:2	FITC
	CD11c	3.9	1:1	BV421
	CD14	MφP9	1:2	APC-H7
	CD19	SJ25C1	1:2	PerCP-Cy5.5
	CD16	3G8	1:2	PE-Cy7
	CD56	NCAM16.2	1:1	PE
	CD123	9F5	1:1	BV510

* All antibodies were manufactured by BD Biosciences. APC = allophycocyanin; BB = Brilliant Blue™; BV = Brilliant Violet™; Cy = cyanine; FITC = fluorescein-isothiocyanate; PE = phycoerythrin; PerCP = peridinin-chlorophyll-protein.

## Data Availability

Raw datasets are available upon request from the authors.

## Supplementary Materials

The following supporting information can be downloaded at https://www.mdpi.com/article/10.3390/biomedicines12102319/s1.
