# Supplementary material for: Flow Cytometric Immunophenotyping: Minimal Differences in Fresh and Cryopreserved Peripheral Blood Mononuclear Cells versus Whole Blood
Source: Biomedicines. 2024 Oct 11;12(10):2319. doi: 10.3390/biomedicines12102319 (PMC11505181; doi:10.3390/biomedicines12102319)
Supplement: Supplementary file 1 [file biomedicines-12-02319-s001.zip › biomedicines-3229144-supplementary.pdf]

Supplementary results

A

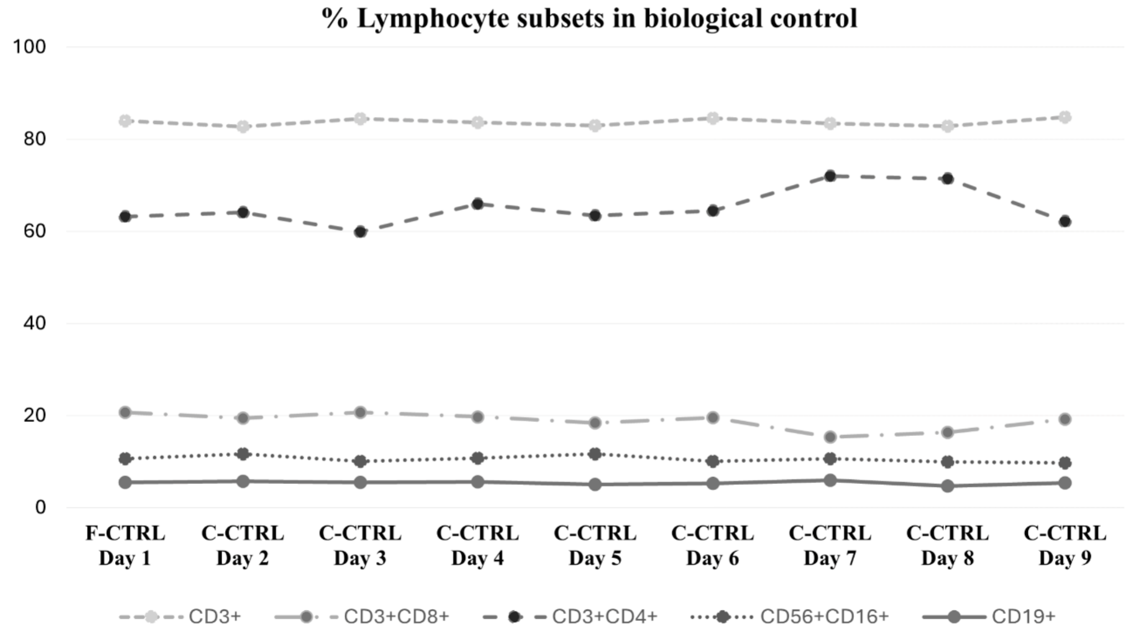

B

	C-CTRL		
	Mean	SD	CV (%)
% CD3+	83.6	0.8	1.0
% CD3+CD8+	18.5	1.8	9.9
% CD3+CD4+	65.4	4.3	6.5
% CD56+CD16+	10.5	0.7	7.0
% CD19+	5.3	0.4	7.5

Supplementary Figure S1

A biological control consisting of PBMCs was analyzed at each experimental flow cytometric run. **A)** The percentages of $CD3^+$ (T), $CD3^+CD4^+$ (Th), $CD3^+CD8^+$ (Tc), $CD19^+$ (B) and $CD56^+CD16^+$ (NK) cells determined in fresh and cryopreserved control PBMCs. **B)** The mean percentage, standard deviation and CV of the cryopreserved biological control. The intra-assay variability for the biological controls is below the acceptable limits (<10%). PBMCs=Peripheral Blood Mononuclear Cells. F-CTRL=Fresh Control PBMCs. C-CTRL=Cryopreserved Control PBMCs.

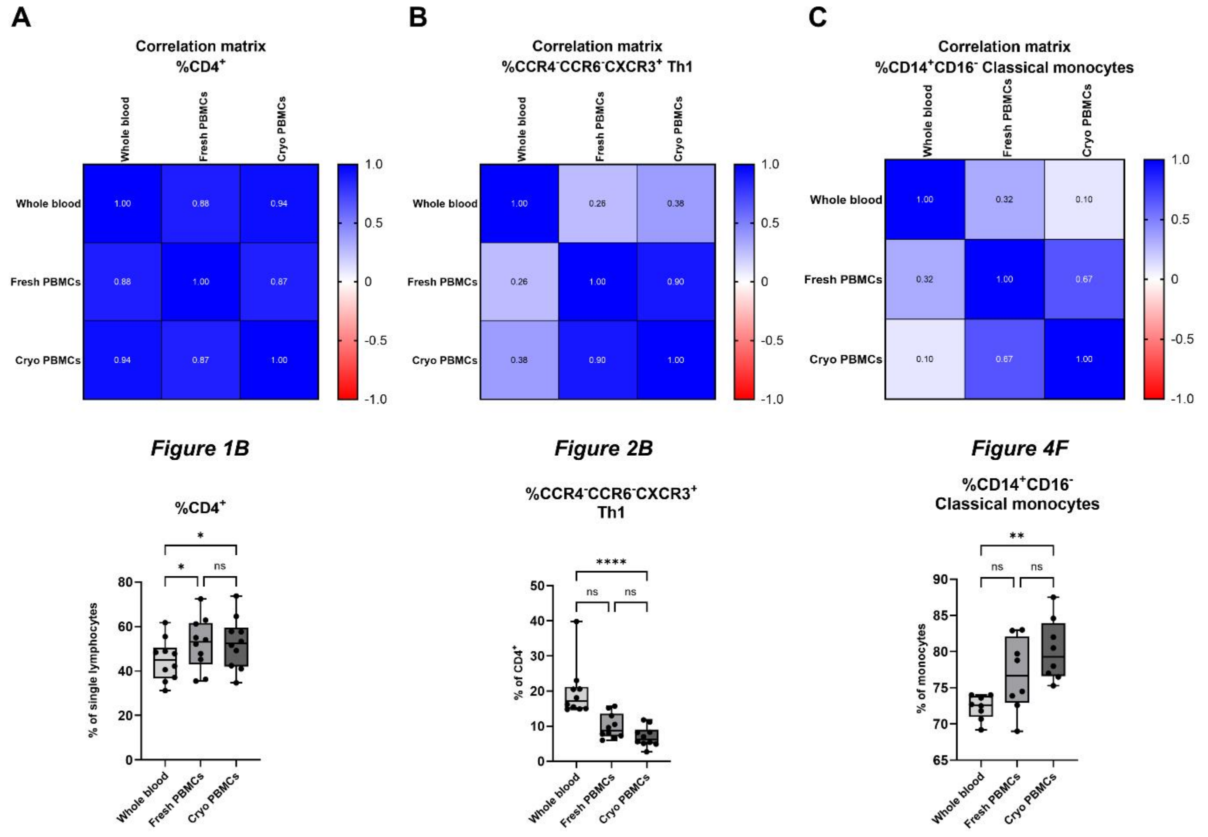

Supplementary Figure S2

Upper row: Matrices with correlation coefficients for the percentage of **A)** CD4$^+$ T helper cells, **B)** Th1-like cells (CCR4$^-$CCR6$^-$CXCR3$^+$) and **C)** classical (CD14$^+$CD16$^-$) monocytes. PBMCs=Peripheral Blood Mononuclear Cells. Cryo=Cryopreserved. MFI=Median Fluorescence Intensity. Paired non-parametric. Non-parametric Spearman correlation, n=10 for **A-B**, for n=8 for **C**. Lower row: Figures 1B, 2B and 4F from the main manuscript to facilitate understanding of the correlation matrices.

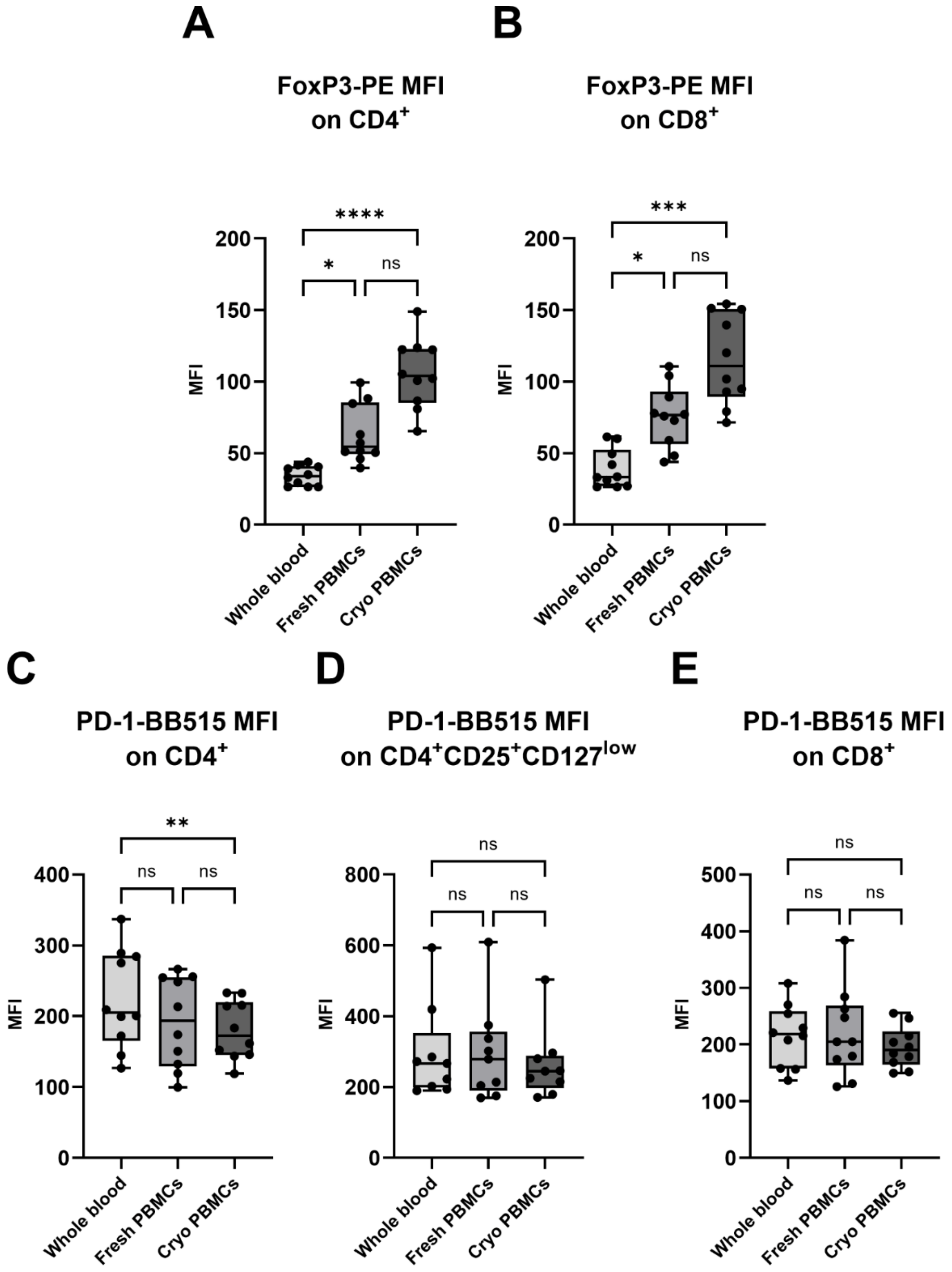

Supplementary Figure S3

MFI values for FoxP3 on **A)** $CD4^+$ T helper cells and on **B)** $CD8^+$ cytotoxic T cells, and for PD-1- on **C)** $CD4^+$, **D)** $CD4^+CD25^+CD127^{low}$ and **E)** $CD8^+$ T cells as measured by flow cytometry. PBMCs=Peripheral Blood Mononuclear Cells. Cryo=Cryopreserved. MFI=Median Fluorescence Intensity. ns=non-significant. Paired non-parametric Friedman test followed by Dunn's multiple comparisons test, n=10 for **A-C** and **E**, n=9 for **D**. *p<0.05, ** p<0.01, *** p<0.001, **** p<0.0001 (adjusted p-values).

Supplementary Table S1

MFI values for various markers on $CD4^{+}$ T helper and $CD8^{+}$ cytotoxic T cell subpopulations.

PBMCs=Peripheral Blood Mononuclear Cells. MFI=Median Fluorescence Intensity. Paired non-parametric Friedman test followed by Dunn's multiple comparisons test, n=10 for all populations except for $CD4^{+}CD25^{+}CD127^{low}$ where n=9.

Statistically significant indicates p<0.05 (adjusted p-values).

Data show median MFI and range (min-max).

		CD26 MFI	CD39 MFI	CD122 MFI	CD127 MFI	CCR4 MFI	CCR5 MFI	CCR6 MFI	CCR10 MFI	CXCR3 MFI	Perforin MFI
**CD4+**	Whole blood	1277 (495-2055)	N/A	26 (26-56)	3899 (2957-5812)	26 (26-26)	26 (26-181)	703 (400-1787)	186 (162-445)	26 (26-322)	N/A
	Fresh PBMCs	1186 (204-1788)$^{a\downarrow c\downarrow}$		36 (26-67)$^{a\uparrow}$	2942 (100-4248)	32 (26-78)$^{a\uparrow}$	26 (26-26)	532 (386-977)	120 (84-204)$^{a\downarrow}$	77 (51-157)$^{a\uparrow}$	
	Cryopreserved PBMCs	1333 (587-2221)		29 (26-64)	1693 (1347-2939)$^{a\downarrow}$	27 (26-34)$^{a\uparrow}$	26 (26-41)	369 (257-1105)$^{a\downarrow}$	117 (86-145)$^{a\downarrow}$	62 (34-119)	
**CD4+CD25+CD127low**	Whole blood	759 (552-979)	N/A	149 (108-172)	849 (662-1096)	34 (26-179)	N/A	934 (553-2938)	N/A	28 (26-209)	N/A
	Fresh PBMCs	637 (368-845)		168 (113-219)	655 (454-865)	57 (26-70)		665 (510-959)		88 (50-147)$^{a\uparrow}$	
	Cryopreserved PBMCs	679 (416-930)		150 (120-169)	368 (315-765)$^{a\downarrow b\downarrow}$	33 (26-66)$^{b\downarrow}$		527 (365-1009)$^{a\downarrow}$		61 (41-109)	
**CD8+**	Whole blood	459 (312-1416)	128 (113-139)	295 (193-817)	2225 (674-4765)	N/A	327 (27-1307)	N/A	211 (178-438)	N/A	1130 (388-3568)
	Fresh PBMCs	434 (282-1323)	134 (117-142)	198 (120-328)$^{a\downarrow}$	1124 (147-2809)		228 (104-374)		233 (123-418)		735 (525-3228)
	Cryopreserved PBMCs	628 (373-1361)	133 (118-148)	209 (130-391)	906 (466-2176)$^{a\downarrow}$		238 (140-318)$^{a\downarrow}$		210 (118-270)		1056 (522-8082)$^{a\downarrow}$
**CD8+ Naive/Stem cell memory**	Whole blood	N/A	122 (106-150)	N/A	N/A	N/A	N/A	N/A	N/A	N/A	230 (150-294)
	Fresh PBMCs		133 (111-155)$^{a\uparrow}$								347 (221-766)$^{a\uparrow}$
	Cryopreserved PBMCs		145 (108-167)								432 (356-849)$^{a\uparrow}$
**CD8+ Central memory**	Whole blood	N/A	143 (126-166)	N/A	N/A	N/A	N/A	N/A	N/A	N/A	299 (196-463)
	Fresh PBMCs		148 (131-157)								419 (269-895)
	Cryopreserved PBMCs		152 (126-172)								494 (381-952)$^{a\uparrow}$
**CD8+ Effector memory**	Whole blood	N/A	132 (114-147)	N/A	N/A	N/A	N/A	N/A	N/A	N/A	1449 (383-4624)
	Fresh PBMCs		136 (129-153)								3085 (562-5578)$^{a\uparrow}$
	Cryopreserved PBMCs		136 (116-162)								2434 (716-5395)$^{a\uparrow}$
**CD8+ TEMRA**	Whole blood	N/A	119 (91-134)	N/A	N/A	N/A	N/A	N/A	N/A	N/A	8753 (2295-26427)
	Fresh PBMCs		122 (96-133)								15671 (2682-35860)
	Cryopreserved PBMCs		116 (101-148)								11260 (2362-37436)

Statistically significant compared with **a.** Whole blood, **b.** Fresh PBMCs, **c.** Cryopreserved PBMCs.

↑ indicates a higher MFI, ↓ indicates a lower MFI

Supplementary Table S2

MFI values for various markers on $CD19^+$ B cells and monocyte subpopulations.

PBMCs=Peripheral Blood Mononuclear Cells. MFI=Median Fluorescence Intensity.

Paired non-parametric Friedman test followed by Dunn's multiple comparisons test, n=8.

Statistically significant indicates p<0.05 (adjusted p-values).

Data show median MFI and range (min-max).

		CD1a MFI	CD11c MFI	CD123 MFI
**B cells**	Whole blood	26 (26-147)	90 (70-126)[b↓]	223 (145-811)[a↑b↑]
	Fresh PBMCs	26 (26-26)	486 (76-697)[a↑]	136 (28-715)[a↓]
	Cryopreserved PBMCs	26 (26-26)	404 (205-539)	121 (26-575)[a↓]
**CD14+CD16- Classical monocytes**	Whole blood	26(26-126)	1695 (786-3902)[b↓]	376 (313-741)[a↑b↑]
	Fresh PBMCs	26 (26-271)	23568 (846-41848)[a↑]	26 (26-646)[a↓]
	Cryopreserved PBMCs	26 (26-26)	23664 (9534-33756)	26 (26-26)[a↓]
**CD14+CD16+ Intermediate monocytes**	Whole blood	26 (26-233)	3435 (1666-7039)[b↓c↓]	842 (358-2958)[a↑b↑]
	Fresh PBMCs	124 (26-401)	44176 (1958-67688)[a↑]	26 (26-2916)[a↓]
	Cryopreserved PBMCs	74 (26-154)	37301 (8130-70665)[a↑]	26 (26-26)[a↓]
**CD14-CD16+ Nonclassical monocytes**	Whole blood	42 (26-205)	1059 (462-1875)[b↓]	1178 (503-4807)[a↑b↑]
	Fresh PBMCs	58 (26-84)	20304 (530-35816)[a↑]	26 (26-4277)[a↓]
	Cryopreserved PBMCs	43 (29-75)	15938 (3782-34718)	26 (26-2651)[a↓]

Statistically significant compared with **a.** Whole blood, **b.** Fresh PBMCs, **c**. Cryopreserved PBMCs.

↑ indicates a higher MFI, ↓ indicates a lower MFI
